# Adjuvant chemotherapy is not a decisive factor in improving the overall survival of pulmonary sarcoma: A population-based study

**DOI:** 10.3389/fonc.2022.940839

**Published:** 2022-08-25

**Authors:** Long Liang, Zixuan Liu, Changhui Wang, Shuanshuan Xie

**Affiliations:** ^1^ Department of Respiratory Medicine, Shanghai Tenth People’s Hospital, Tongji University School of Medicine, Shanghai, China; ^2^ Tongji University School of Medicine, Shanghai, China

**Keywords:** pulmonary sarcomatoid carcinoma, non-small-cell lung cancer, adjuvant chemotherapy, overall survival, risk factors

## Abstract

**Objective:**

This study aimed to investigate the impact of adjuvant chemotherapy on overall survival (OS) for pulmonary sarcomatoid carcinoma (PSC) and non-small-cell lung cancer (NSCLC) cohorts and to identify its potential risk factors.

**Methods:**

A retrospective analysis was performed by querying the Surveillance, Epidemiology, and End Results (SEER) database for patients diagnosed as having PSC (n=460) and NSCLC (n=140,467) from 2004 to 2015. The demographics, tumor characteristics, treatment modes, and survival were included in the scope of statistical analysis. Confounding factors were controlled by propensity score matching (PSM) analysis. Kaplan–Meier survival curves were performed to compare the effects of adjuvant chemotherapy on OS of the patients in the two cohorts (PSC *vs*. NSCLC). A multivariable Cox regression model was constructed, and Kaplan–Meier analysis on each variate was applied to predict risk factors associated with OS.

**Results:**

When adjuvant chemotherapy approach was applied in the treatment of patients with PSC or adjusted NSCLC, respectively, an improved OS could be observed in the NSCLC cohort (p=0.017). For the entire PSC cohort, 1-, 3-, and 5-year OS were 25.43%, 13.04%, and 6.96%, respectively, compared with 41.96%, 17.39%, and 10.00%, respectively, for the new adjusted NSCLC cohort after PSM, which were statistically significant difference (p<0.001). Multivariable Cox regression analysis was performed on OS covering prognostic factors such as primary site (p=0.036), first malignant indicator (p<0.001), age at diagnosis (p<0.001), marital status at diagnosis (p=0.039), and high school education (p=0.045). Additionally, patients with the following parameters had the worse impact on OS: a poorly differentiated pathology (Grade III/IV, p=0.023), older age (p<0.001), liver or lung metastasis (p=0.004, p=0.029), and the number of lymph nodes removed <4 (p<0.001).

**Conclusions:**

Adjuvant chemotherapy did not play a decisive role in improving the OS of PSC, while it was associated with improved OS of NSCLC.

## Introduction

Pulmonary sarcomatoid carcinoma (PSC) is associated with the characteristics of rarity and more aggressive behavior in all non-small-cell lung cancer (NSCLC) subtypes, which accounts for 0.1%–0.4% of all lung malignancy (1). Compared with other subtypes of NSCLC, the clinical symptoms and classical morphology of PSC are non-specific to distinguish. Current reports on these tumors are mostly limited to the clinical data with small sample size and retrospective analysis extracted from shared databases (2, 3).

Due to the traits of easy invasion and distant metastasis, patients with PSC typically have a poor prognosis even in the early stages of the disease (4). A study using the National Cancer Database (NCDB) reported that PSC is significantly associated with worse survival outcomes compared with conventional NSCLC (5), and the significance of this contrasting survival curve exists across all stages of the disease. The American Cancer Society estimates that the 5-year survival rate of PSC is only 15%–20.1% (6). To date, there are no consensus on guiding PSC patients for standard management strategies. Surgical resection is considered a feasible and effective treatment modality for this rare cancer. However, the benefit of adjuvant chemotherapy pre-/post-operative is still controversial (7–9).

At present, there are few clinical studies on the survival outcomes and prognostic factors for PSC, leading to the treatment regime not fully figured out. We investigated whether adjuvant chemotherapy played a positive role on overall survival (OS) for PSC and NSCLC cohorts. Kaplan–Meier survival curves and multivariable Cox proportional hazard analysis were used to screen for risk factors with an impact on OS. This knowledge will be useful to better understand the progression, prevention, and treatments in PSC disease.

## Methods

### Data source

The Surveillance, Epidemiology, and End Results (SEER) database is a large tumor database established by the National Cancer Institute (http://seer.cancer.gov/), which records the incidence, mortality, and prevalence of millions of cancer patients in the United States. The data that we utilized were derived from the SEER database. The dataset includes a detailed patient information such as basic demographic characteristics, survival time, treatment mode, distribution of the lesion, pathological type, and degree of differentiation. The content of this study complies with the relevant provisions of the Declaration of Helsinki, which establishes the ethical principles concerning medical research on human subjects, is a limitation for biomedical research involving people as subjects, and is the second international document on human trials, which is more comprehensive, concrete, and perfect than the Nuremberg Code.

### Study population

**Figure 1:**
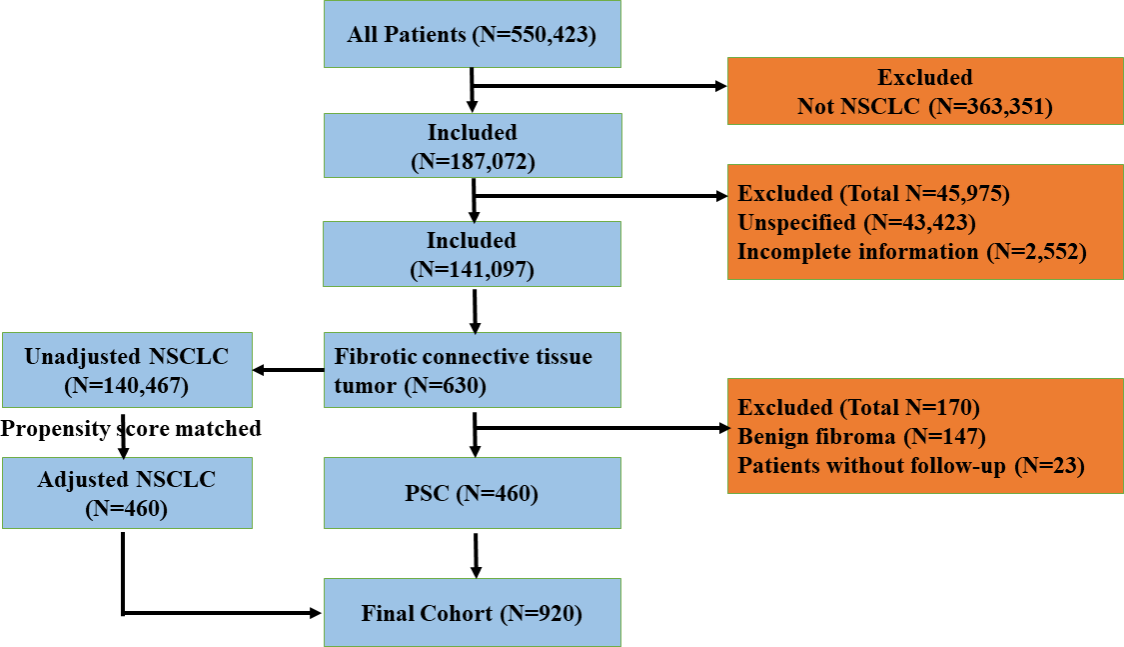
The flowchart of exclusion criteria and study design. PSC, pulmonary sarcomatoid carcinoma; NSCLC, non-small-cell lung cancer.

We extracted lung-cancer-related data from the SEER database for a retrospective analysis study; the detailed screening flowchart is shown in **Figure 1**. We set the filtering conditions for the cohorts pathologically diagnosed with NSCLC and PSC from 2004 to 2015. NSCLC (squamous cell carcinoma, adenocarcinoma, large cell carcinoma, and others) was chosen as the comparator for PSC (giant-cell carcinoma, small-cell carcinoma, epithelioid carcinoma, undifferentiated carcinoma, and desmoplastic carcinoma), as these tumors are morphologically indistinguishable.

### Data elements

Our study aimed to explore the impact of adjuvant chemotherapy on the 1-, 3-, and 5-year survival rates of PSC and NSCLC cohorts and to identify the independent prognostic factors that have an impact on OS, which was defined as the time period from the diagnosis of the disease to the date of death. A complete list of data information on patients are available online. The effect of each covariate on OS was analyzed independently, including race, sex, age at diagnosis, regional distribution, primary site of lesions, tumor grade, laterality, histopathological subtype, the number of lymph nodes surgical removed, radiation, chemotherapy, bone/brain/liver/lung metastasis, first malignant indicator, insurance status, marital status, high school education, and median household income.

### Statistical analysis

All the data in this study were analyzed by using IBM SPSS 25.0 version (IBM Corp, Armonk, NY, USA). The chi-square test was applied for the analysis of categorical variables. In the univariate analysis, a non-parametric test was used to detect the effect of each variable on OS. A multivariable Cox proportional hazards regression model was constructed to further determine the independent predictors of survival. The method of log-rank test was used for the comparison of the survival curves. Kaplan–Meier survival curves were created to compare survival time of the subtypes, which include grade, age at diagnosis, liver metastasis, lung metastasis, and lymph nodes removed. The means of propensity score matching (PSM) was applied to bridge the differences when comparing the survival time between PSC and NSCLC cohorts and to estimate the effect of chemotherapy on these two cohorts. Two-tailed p-values of <0.05 were considered statistically significant.

## Results

### Baseline cohort characteristics

A total of 460 patients in the PSC study group were compared with 140,467 NSCLC patients enrolled in the statistical analysis during the same study period. All patients’ data were extracted from the SEER database. PSC patients, of which 83.0% were white and 63.0% were male, were compared with NSCLC patients, of which 82.3% were white and 60.3% were male, and the proportions of race and gender did not differ between the two cohorts (p=0.421; p=0.233). Notably, a greater fraction of tumors in the PSC and NSCLC cohorts were located in the upper lobe of the lungs. Meanwhile, the proportion of lesions in the upper lung lobe in the PSC cohort was less than that in the NSCLC cohort (p<0.001). Furthermore, patients diagnosed with PSC were more inclined to be younger than 45 years, live in the northwest region, have a poorly differentiated or undifferentiated lesion (Grade III/IV), have laterality to the left, have 0–3 lymph nodes removed, have less selection of radiation and chemotherapy, and have higher levels of median household income compared with the NSCLC cohort of patients. Other demographic variables such as the year of diagnosis, insurance, and marital status of patients showed no statistically significant differences between the two groups. All the data are summarized in **Table 1**.

**Table 1:** Baseline cohort characteristics.

Basic characteristics	PSC (n = 460, %)	NSCLC (n = 140,467, %)	χ^2^	p
**Race**			1.728	0.421
White	382 (83.1)	115,615 (82.3)		
Black	48 (10.4)	16,966 (12.1)		
Others	30 (6.5)	7,886 (5.6)		
**Sex**			1.448	0.233
Male	290 (63.0)	84,693 (60.3)		
Female	170 (37.0)	55,774 (39.7)		
**Age (year)**			118.002	**<0.001**
<45	34 (7.4)	2,126 (1.5)		
≥45, <55	40 (8.7)	9,801 (7.0)		
≥55, <65	90 (19.6)	29,853 (21.3)		
≥65, <75	119 (25.9)	49,078 (34.9)		
≥75	177 (38.5)	49,608 (35.3)		
**Year of diagnosis**			1.262	0.532
2004–2007	159 (34.6)	45,356 (32.3)		
2008–2011	154 (33.5)	47,488 (33.8)		
2012–2015	147 (32.0)	47,622 (33.9)		
**Region**			29.350	**<0.001**
East	173 (37.6)	67,316 (47.9)		
North	43 (9.3)	15,980 (11.4)		
Southwest	15 (3.3)	4,006 (2.9)		
Northwest	229 (49.8)	53,164 (37.8)		
**Primary site**			73.78	**<0.001**
Upper lobe	190 (41.3)	71,481 (50.9)		
Middle lobe	19 (4.1)	5,308 (3.8)		
Lower lobe	125 (27.2)	40,101 (28.5)		
NOS	95 (20.7)	13,617 (9.7)		
Overlapping lesion	12 (2.6)	1,954 (1.4)		
Main bronchus	17 (3.7)	7,693 (5.5)		
Trachea	2 (0.4)	312 (0.2)		
**Grade**			1,560.434	**<0.001**
Grade I	8 (1.7)	7,429 (5.3)		
Grade II	15 (3.3)	33,911 (24.1)		
Grade III	106 (23.0)	42,419 (30.2)		
Grade IV	147 (32.0)	3,669 (2.6)		
Unknown	184 (40.0)	53,038 (37.8)		
**Laterality**			11.498	**0.009**
Right	228 (49.6)	77,507 (55.2)		
Left	207 (45.0)	57,997 (41.3)		
Bilateral	11 (2.4)	1,573 (1.1)		
Others	14 (3.0)	3,389 (2.4)		
**Lymph nodes removed**			27.893	**<0.001**
0–3 lymph nodes removed	367 (79.8)	103,359 (73.6)		
≥4 lymph nodes removed	64 (13.9)	27,174 (19.3)		
Regional biopsy or aspiration	7 (1.5)	6,371 (4.5)		
Sentinel lymph nodes biopsy	2 (0.4)	260 (0.2)		
Others	20 (4.3)	3,302 (2.4)		
**Radiation**			235.141	**<0.001**
Beam radiation	120 (26.1)	84,385 (60.1)		
Rad not specified	3 (0.7)	709 (0.5)		
Unknown	333 (72.4)	53,113 (37.8)		
Refused	4 (0.9)	1,837 (1.3)		
Beam with plants or isotopes	0 (0.0)	1,91 (0.1)		
Implants or isotopes	0 (0.0)	231 (0.2)		
**Chemotherapy**			43.545	**<0.001**
No	325 (70.7)	77,724 (55.3)		
Yes	135 (29.3)	62,742 (44.7)		
**First malignant indicator**			18.485	**<0.001**
No	147 (32.0)	32,934 (23.4)		
Yes	313 (68.0)	107,532 (76.6)		
**Insurance status**			3.263	0.353
Medicaid	43 (9.3)	14,432 (10.3)		
Insured or no specifics	273 (59.3)	87,231 (62.1)		
Uninsured	9 (2.0)	2,632 (1.9)		
Blanks or unknown	135 (29.3)	36,171 (25.8)		
**Marital status**			4.663	0.097
Married or domestic partner	256 (55.7)	72,121 (51.3)		
Divorced or separated or single or windowed	182 (39.6)	62,543 (44.5)		
Unknown=3	22 (4.8)	5,802 (4.1)		
**High school education (Score)**			11.390	**0.01**
≤1,000	78 (17.0)	28,284 (20.1)		
1,000–2,000	239 (52.0)	72,424 (51.6)		
2,000–3,000	137 (29.8)	35,283 (25.1)		
>3,000	6 (1.3)	4,475 (3.2)		
**Median household income ($/month)**			11.729	**0.008**
≤5,000	43 (9.3)	18,426 (13.1)		
>5,000, ≤7,000	222 (48.3)	68,110 (48.5)		
>7,000, ≤9,000	147 (32.0)	36,887 (26.3)		
>9,000	48 (10.4)	17,043 (12.1)		

PSC, pulmonary sarcomatoid carcinoma; NSCLC, non-small-cell lung cancer.

A p-value of <0.05 represents a significant statistical difference.

Bold indicate p values < 0.05 are statistically significant.

### A univariate survival analysis in PSC cohort

We identified each covariate such as primary site (χ^2^ = 16.648, p=0.023), radiation (χ^2^ = 11.366, p=0.01), chemotherapy (χ^2^ = 24.171, p<0.001), bone metastasis (χ^2^ = 6.202, p=0.045), liver metastasis (χ^2^ = 6.202, p=0.045), lung metastasis (χ^2^ = 9.314, p=0.009), first malignant indicator (χ^2^ = 8.504, p=0.004), age at diagnosis (χ^2^ = 28.230, p<0.001), and marital status at diagnosis (χ^2^ = 10.773, p=0.005) and were shown to be significantly associated with OS by adopting the method of non-parametric test analysis, while race (χ^2^ = 1.186, p=0.553), sex (χ^2^ = 0.734, p=0.392), region (χ^2^ = 1.015, p=0.798), grade (χ^2^ = 8.444, p=0.077), laterality (χ^2^ = 4.025, p=0.259), histological type (χ^2^ = 5.141, p=0.526), brain metastasis (χ^2^ = 4.256, p=0.119), insurance state (χ^2^ = 5.193, p=0.158), high school education (χ^2^ = 6.778, p=0.079), and median family income (χ^2^ = 4.319, p=0.229) were not significantly associated with OS in PSC cohort (as summarized in **Supplementary Table S1**).

A total of 11 factors comprising primary site, grade, radiation, chemotherapy, bone/liver/lung metastasis, first malignant indicator, age at diagnosis, marital status, and high school education were screened out in utilizing univariate survival analysis (p<0.1). Next, a multivariable Cox regression analysis model was constructed to further evaluate the independent risk factors on OS.

### Multivariable Cox proportional hazards analysis of OS in PSC cohort

Within the multivariable Cox proportional hazards analysis, important prognostic factors for OS constitute of primary site (p=0.036), first malignant indicator (p<0.001), age at diagnosis (p<0.001), marital status at diagnosis (p=0.039), and high school education (p=0.045). The predictors of OS by Cox regression analysis did not include grade (p=0.061), radiation (p=0.507), chemotherapy (p=0.260), bone metastasis (p=0.255), liver metastasis (p=0.091), and lung metastasis (p=0.309). Several covariates such as age, marital status, and high school education were further stratified for survival analysis; patients over 55 years old remained independently associated with lower OS compared with younger patients (HR of 1.725, 95% CI 1.040–2.861, p=0.035 for age ≥55; HR of 2.233, 95% CI 1.376–3.624, p=0.001 for age ≥65; HR of 3.053, 95% CI 1.889–4.936, p<0.001 for age ≥75). Being divorced, separated, singled, or windowed was independently associated with lower OS compared with being married or having a domestic partner (HR, 1.337; 95% CI, 1.069–1.672, p=0.011). Patients who received higher school education were more likely to have PSC compared with those who received lower school education (HR, 1.605; 95% CI, 1.151–2.238, p=0.005) (as summarized in **Table 2**).

**Table 2:** Multivariable Cox proportional hazards analysis of OS in PSC cohort.

Covariate	HR	95% CI	p
**Primary site**			**0.036**
Upper lobe	Reference		
Middle lobe	1.227	0.721 – 2.089	0.452
Lower lobe	0.967	0.745 – 1.256	0.803
NOS	1.539	1.158 – 2.044	0.003
Overlapping lesion	1.186	0.606 – 2.320	0.618
Main bronchus	0.751	0.408 – 1.384	0.359
Trachea	0.654	0.085 – 5.015	0.683
**Grade**			0.061
Grade I	Reference		
Grade II	1.421	0.431 – 4.682	0.564
Grade III	2.746	0.988 – 7.629	0.053
Grade IV	2.523	0.916 – 6.952	0.073
Unknow	2.104	0.765 – 5.784	0.149
**Radiation**			0.507
Beam radiation	Reference		
Not specified	0.352	0.085 – 1.455	0.149
Unknown	0.961	0.722 – 1.279	0.784
Refused	0.716	0.214 – 2.395	0.587
**Chemotherapy**			0.260
No	Reference		
Yes	0.869	0.680 – 1.110	0.260
**Bone metastasis**			0.255
No	Reference		
Yes	1.527	0.905 – 2.575	0.113
Others	1.395	0.474 – 4.109	0.546
**Liver metastasis**			0.091
No	Reference		
Yes	2.129	1.018 – 4.454	**0.045**
Others	0.535	0.127 – 2.248	0.393
**Lung metastasis**			0.309
No	Reference		
Yes	1.407	0.906 – 2.187	0.129
Others	1.375	0.337 – 5.603	0.657
**First malignant indicator**			**<0.001**
No	Reference		
Yes	1.543	1.219 – 1.952	**<0.001**
**Age at diagnosis(year)**			**<0.001**
<45	Reference		
≥45, <55	1.748	0.984 – 3.106	0.057
≥55, <65	1.725	1.040 – 2.861	**0.035**
≥65, <75	2.233	1.376 – 3.624	**0.001**
≥75	3.053	1.889 – 4.936	**<0.001**
**Marital status**			**0.039**
Married or domestic partner	Reference		
Divorced or separated or single or windowed	1.337	1.069 – 1.672	**0.011**
Unknown	1.140	0.673 – 1.930	0.626
**High school education** **(score)**			**0.045**
≤1,000	Reference		
1,000 – 2,000	1.379	1.015 – 1.872	**0.040**
2,000 – 3,000	1.605	1.151 – 2.238	**0.005**
>3,000	1.076	0.418 – 2.768	0.879

PSC, pulmonary sarcomatoid carcinoma; OS, overall survival; HR, hazard ratio; CI, confidence interval; NOS, not otherwise specified.

A p-value <0.05 represents a significant statistical difference.

Bold indicate p values < 0.05 are statistically significant.

### Kaplan–Meier analysis of survival curves between the PSC and NSCLC cohorts

**Figure 2:**
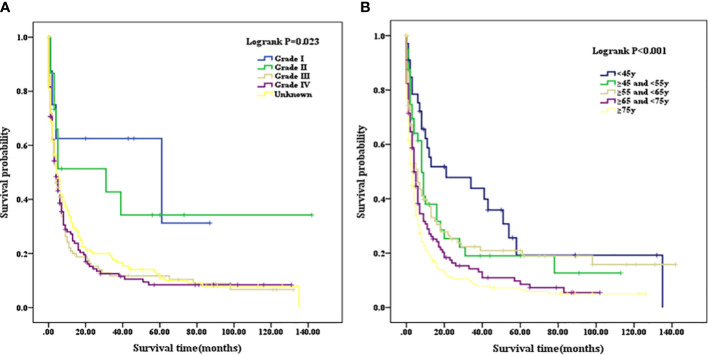
**(A)** Kaplan–Meier analysis of survival curves stratified by grade in PSC cohort. **(B)** Kaplan–Meier analysis of survival curves stratified by age stages in PSC cohort. PSC, pulmonary sarcomatoid carcinoma.

**Figure 3:**
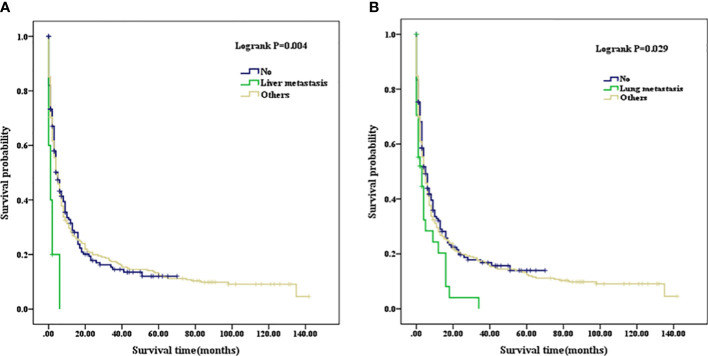
**(A)** Kaplan–Meier analysis of survival curves stratified by liver metastasis in PSC cohort. **(B)** Kaplan–Meier analysis of survival curves stratified by lung metastasis in PSC cohort. PSC, pulmonary sarcomatoid carcinoma.

**Figure 4:**
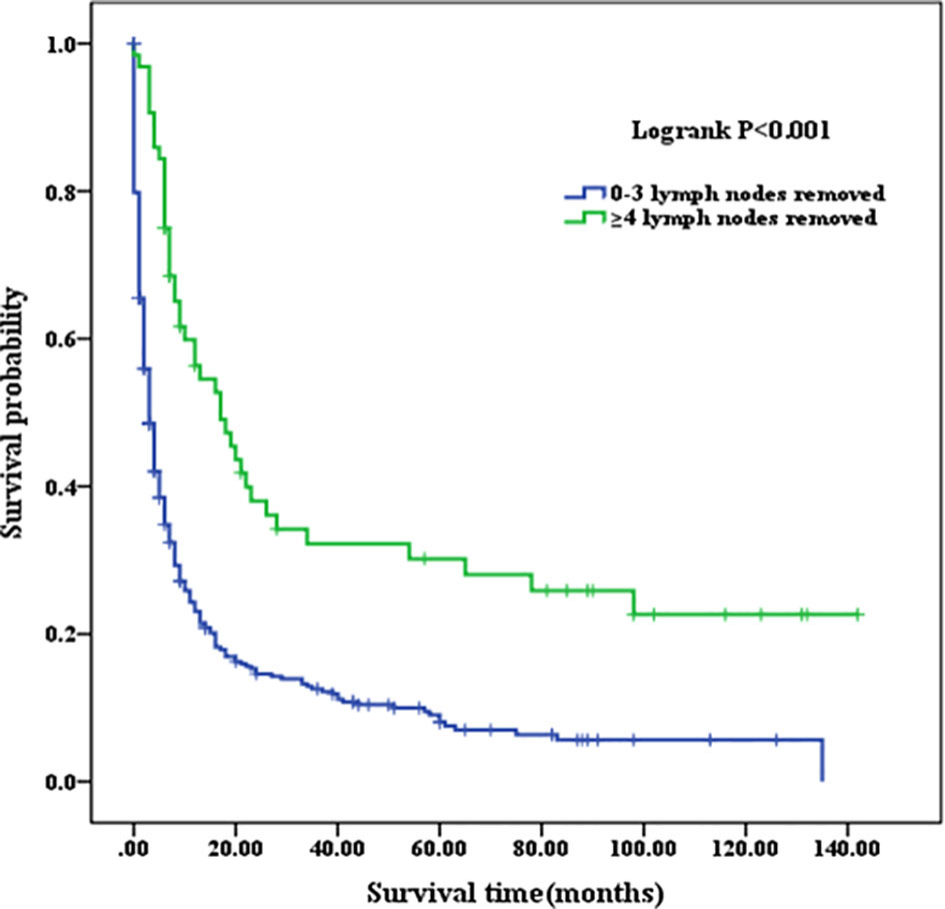
Kaplan–Meier analysis of survival curves stratified by the number of lymph node removed in PSC cohort. PSC, pulmonary sarcomatoid carcinoma.

Patients with a well or moderately differentiated PSC (Grade I/II) had better OS compared with those with a poorly differentiated or undifferentiated lesion (Grade III/IV) (p=0.023, as shown in **Figure 2A**). Among patients of different age stages, younger subjects clearly have a longer OS compared with older individuals (p<0.001, as shown in **Figure 2B**). Patients with liver or lung metastasis were closely associated with inferior OS compared with those without metastasis (p=0.004, p=0.029, respectively, as shown in **Figure 3**). Patients with four or more lymph nodes removed have improved OS compared with those managed with zero to three lymph nodes removed (p<0.001, as shown in **Figure 4**).

For the entire cohort in PSC, 1-, 3-, and 5-year OS were 25.43%, 13.04%, and 6.96%, respectively; when calculated according to differentiated grades, OS were 47.83%, 3.91%, and 4.35% for grade I/II, respectively, and 20.55%, 9.49%, and 6.72% for grade III/IV, respectively. The 3- and 5-year OS in grade III/IV were significantly higher than that in grade I/II (p=0.006). For the entire cohort in unadjusted NSCLC, 1-, 3-, and 5-year OS were 40.70%, 16.38%, and 8.66% respectively, which were significantly higher than that in the PSC cohort (p<0.001). A new NSCLC cohort was created after PSM with PSC cohort. We calculated that the 1-, 3-, and 5-year OS were 41.96%, 17.39%, and 10.00%, respectively, for the new adjusted NSCLC cohort, which were also significantly higher than the PSC cohort (p<0.001) (as summarized in **Table 3**).

**Table 3:** Comparison of 1, 3, and 5-year OS between the PSC and NSCLC cohorts.

PLS	1-year OS (n/N, %)	3-year OS (n/N, %)	5-year OS (n/N, %)	p
Overall	117/460 (25.43)	60/460 (13.04)	32/460 (6.96)	**0.006**
Grade I/II	11/23 (47.83)	9/23 (3.91)	1/23 (4.35)	
Grade III/IV	52/253 (20.55)	24/253 (9.49)	17/253 (6.72)	
Unknown	54/184 (29.35)	27/184 (14.67)	14/184 (7.61)	
**Unadjusted NSCLC**	57,172/140,467 (40.70)	23,010/140,467 (16.38)	12,160/140,467 (8.66)	**<0.001**
**Adjusted NSCLC**	193/460 (41.96)	80/460 (17.39)	46/460 (10)	**<0.001**

PSC, pulmonary sarcomatoid carcinoma; NSCLC, non-small-cell lung cancer; OS, overall survival.

A p-value of <0.05 represents a significant statistical difference.

Bold indicate p values < 0.05 are statistically significant.

### Comparison of median survival time and adjuvant chemotherapy between the PSC and NSCLC cohorts

**Figure 5:**
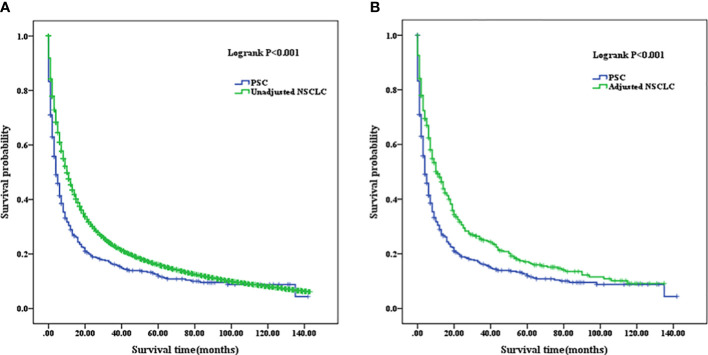
**(A)** Comparison of Kaplan–Meier analysis of survival curves between PSC and unadjusted NSCLC cohorts. **(B)** Comparison of Kaplan–Meier analysis of survival curves between PSC and adjusted NSCLC cohorts. PSC, pulmonary sarcomatoid carcinoma; NSCLC, non-small-cell lung cancer.

The mean OS of PSC was 21.549 months (95% CI, 17.536–25.562), and the median OS of the patients was 4 months (95% CI, 3.034–4.966). The mean OS of unadjusted NSCLC was 28.599 months (95% CI, 28.353–28.844), and the median OS was 10 months (95% CI, 9.888–10.112). The mean OS of adjusted NSCLC was 29.913 months (95% CI, 25.668–34.159), and the median OS was 11 months (95% CI, 8.698–13.302) (as summarized in **Table 4**). OS in unadjusted NSCLC was significantly higher than that in PSC patients (p<0.001); the same comparable trends were also presented after the NSCLC cohort was adjusted (p<0.001). Survival curves are shown in **Figure 5**.

**Table 4:** Comparison of mean and median survival time between the PSC and NSCLC cohorts.

Groups	Mean OS (months)	95%CI		Median OS (months)	95%CI
PLS	21.549		17.536 – 25.562	4.0	3.034 – 4.966
Unadjusted NSCLC	28.599		28.353 – 28.844	10.0	9.888 – 10.112
adjusted NSCLC	29.913		25.668 – 34.159	11.0	8.698 – 13.302

PSC, pulmonary sarcomatoid carcinoma; NSCLC, non-small cell lung cancer; OS, overall survival; CI, confidence interval.

**Figure 6:**
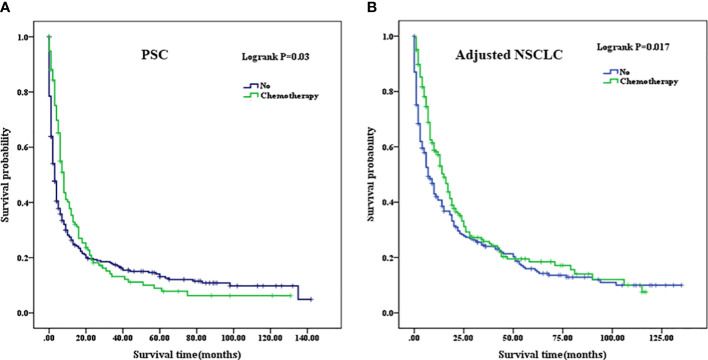
**(A)** Comparison of Kaplan–Meier analysis of survival curves after adjuvant chemotherapy in PSC cohort. **(B)** Comparison of Kaplan–Meier analysis of survival curves after adjuvant chemotherapy in adjusted NSCLC cohort. PSC, pulmonary sarcomatoid carcinoma; NSCLC, non-small-cell lung cancer.

The mean OS of the patients who did not receive chemotherapy in the PSC cohort was 21.990 months (95% CI, 16.982–27.000), and the median OS was 3 months (95% CI, 2.298–3.702) compared with the patients who received chemotherapy, whose mean OS was 19.921 months (95% CI, 13.918–25.924) and median OS was 8 months (95% CI, 6.805–9.195). In the adjusted NSCLC cohort, the mean OS was 27.886 months (95% CI, 22.343–33.428), and the median OS was 7 months (95% CI, 4.929–9.071) for the patients who did not receive chemotherapy compared with the patients who received chemotherapy whose mean OS was 30.793 months (95% CI, 24.974–36.612) and median OS was 15 months (95% CI, 12.329–17.671) (as summarized in **Table 5**). When chemotherapy was applied in the treatment of patients with PSC, there was no improved OS compared with those who did not receive chemotherapy (p=0.03, **Figure 6A**). We considered that the main reason for this phenomenon was that the proportion of patients with well- and moderately differentiated pathological types was low, and chemotherapy can have a better positive therapeutic effect for the above pathological types, while it may have a negative effect on poorly differentiated or undifferentiated pathological types. As a comparison cohort, when chemotherapy was applied in the treatment of patients with adjusted NSCLC, there was statistically significant improvement on OS compared with those patients who did not receive chemotherapy (p=0.017, **Figure 6B**).

**Table 5:** Comparison of mean and median survival time after adjuvant chemotherapy between the PSC and NSCLC cohorts.

Groups	Chemotherapy	Mean OS (months)	95%CI	Median OS (months)	95%CI
PLS	No	21.990	16.982 – 27.000	3	2.298 – 3.702
	Yes	19.921	13.918 – 25.924	8	6.805 – 9.195
	Overall	21.549	17.536 – 25.562	4	3.034 – 4.966
Adjusted NSCLC	No	27.886	22.343 – 33.428	7	4.929 – 9.071
	Yes	30.793	24.974 – 36.612	15	12.329 – 17.671
	Overall	29.913	25.668 – 34.159	11	8.698 – 13.302

PSC, pulmonary sarcomatoid carcinoma; NSCLC, non-small cell lung cancer; OS, overall survival; CI, confidence interval.

## Discussion

We exploited the SEER database to systematically study the impact of clinicopathological characteristics and treatment modalities on the OS of 460 patients with PSC, and a multivariable Cox regression model was constructed to further explore the risk factors for OS. Our results demonstrated that patients with PSC are associated with a higher incidence in younger patients (<45 years), are more likely to live in the northwest, and have a poorly differentiated or undifferentiated lesion compared with the NSCLC cohort of patients. In order to reduce the statistical differences of survival time between the PSC and NSCLC cohorts, an analysis of PSM was performed. A poorer prognosis of patients with PSC was clearly shown compared with that of their NSCLC counterparts by using a well-matched group. Another study (10) also proved a similar outcome in patients with PSC when compared with other NSCLC patients.

Multivariate Cox regression was performed to further analyze risk factors associated with OS. Variables, including primary site, first malignant indicator, age at diagnosis, marital status, and high school education, were independent predictors for OS in patients with PSC. As an independent risk factor, the influence of age at diagnosis on OS has been investigated in previous research (11). Studies have reported that patients with advanced age may have a high likelihood of poor prognosis and increased risk of mortality (12, 13). In our research, age beyond 55 years old was also demonstrated to act as an independent risk factor for OS. Histologically identified lung cancer in younger patients typically shows advanced tumor stages, with more symptoms (14, 15). A recent SEER database examined the effect of age on lung cancer patients, with better overall and cancer-specific survival in younger patients than in the older cohort even though under the condition of presenting with stage IV disease (16). Some community-based and national registries analyze the OS of miscellaneous bronchogenic carcinoma to form the view of improved outcomes in younger cohorts (17, 18). The odds of developing comorbidities achieved a substantial accumulation with increasing age. It has been reported that comorbidity is also an independent predictor affecting patient mortality, which has a direct or indirect impact on OS (19, 20).

At present, surgery seems to be an appropriate choice for PSC treatment (21, 22). Such tumors are often characterized by slow growth and presented at advanced stages when discovered. Endobronchial tumors have a better prognosis compared with peripheral tumors, which are more prone to metastasis and invasion to neighboring tissue structures and the vasculature. After surgery, 1-, 3-, and 5-year survival rates for PSC have been reported in some studies to be worse than those for NSCLC (10, 23, 24). The conclusion is supported in our study as evidenced by the significantly lower 1-, 3-, and 5-year OS compared with those of the NSCLC patients. The number of lymph nodes removed, as an important contributor, has been consistently reported to be associated with prognosis of PSC patients (25–27). The OS of PSC patients with four or more lymph nodes removed was significantly higher compared with that of PSC patients with less than four lymph nodes removed in our analysis. Of course, the location of the lymph node was a prognostic factor for overall survival in lung cancer (28). However, it is a pity that SEER database does not record the details about the station of lymph nodes but instead records the number. Considering the practical application value of this issue, we plan to further analyze the association of the location of the lymph nodes with survival time in PSC in future clinical data acquisition.

Another interesting point of comparison refers to adjuvant chemotherapy being associated with improved OS for NSCLC but not for PSC in the SEER database. One of the main reasons for this phenomenon is that PSC presented with poorly differentiated pathological morphology. These rare, histologically highly malignant tumors have been described to be associated with poor prognosis in relevant literature reports (29, 30). Among many previous studies, numerous papers have verified that adjuvant chemotherapy did not play a positive role in prolonging the OS in the course of the intervention of treatment for PSC (31–33). Nonetheless, a small fraction of studies has shown that some survival benefits could be obtained from adjuvant chemotherapy, which leads to blurred boundaries in physicians’ decision-making regarding whether to take chemotherapy.

Not surprisingly, adjuvant chemotherapy offered different survival benefits in patients with PSC at different stages. When patients with a higher stage disease (stage II and III), the therapeutic effect was particularly pronounced and the OS will be extended after receiving adjuvant chemotherapy compared with the lower stage (stage I). The future directions of pharmacological treatment for PSC might have tendencies toward targeted therapy or immunotherapy (34–36). Undoubtedly, developing a more rational and specific regime plan for each patient instead of general treatment will improve OS of PSC patients (37, 38).

It is also important to consider the potential limitations that affect the analysis of the results in our study. First, a small sample size and the possibility of data bias in this retrospective analysis are difficult to exclude. The finding that PSC patients are more likely to receive a higher degree of school education background and higher levels of salary treatment than NSCLC patients may allude to a referral bias. In addition, the SEER database did not capture the pathological stage for PSC, and therefore, an effective program of staging analysis was not available for all patients. Finally, the specific details on various treatment modalities including chemotherapy regimens were not recorded in SEER database, which comprises the contents of chemotherapeutic drugs, the agents used, biological half-life, toxicity, target genes for the treatment, and the course of taking medication, may impact the final results analysis.

## Conclusions

In conclusion, adjuvant chemotherapy is not an appropriate treatment option for patients with PSC but is certainly effective in patients with NSCLC. Age at diagnosis, an independent risk factor, must be used as an important consideration to weigh whether a chemotherapy regimen should be performed and the dose of chemotherapy drugs. This study also predicted other risk factors affecting OS by building a multivariate regression model, which provides us useful information on prevention and treatment strategies for PSC patients.

## Data availability statement

The datasets presented in this study can be found in online repositories. The names of the repository/repositories and accession number(s) can be found in the article/**Supplementary Material**.

## Author contribution

Conception and design: LL, ZL, SX, and CW. Acquisition, statistical analysis, or interpretation of the data: all authors. Drafting of the manuscript: LL, ZL, SX, and CW. All authors contributed to the article and approved the submitted version.

## Acknowledgments

We would like to thank all the patients who donated their statistical data.

## Conflict of interest

The authors declare that the research was conducted in the absence of any commercial or financial relationships that could be construed as a potential conflict of interest.

## Publisher’s note

All claims expressed in this article are solely those of the authors and do not necessarily represent those of their affiliated organizations, or those of the publisher, the editors and the reviewers. Any product that may be evaluated in this article, or claim that may be made by its manufacturer, is not guaranteed or endorsed by the publisher.

## Supplementary material

Supplementary Table 1 Univariate survival analysis using a non-parametric test on each covariate in PSC cohort. PSC, pulmonary sarcomatoid carcinoma; A p-value of less than 0.05 represents a significant statistical difference.

## Abbreviations

SEER, the Surveillance, Epidemiology, and End Results; OS, overall survival; PSC, pulmonary sarcomatoid carcinoma; NSCLC, non-small cell lung cancer; PSM, propensity score matching.
