# Supplementary material for: Adjuvant chemotherapy is not a decisive factor in improving the overall survival of pulmonary sarcoma: A population-based study
Source: Front Oncol. 2022 Aug 25;12:940839. doi: 10.3389/fonc.2022.940839 (PMC9452885; doi:10.3389/fonc.2022.940839)
Supplement: Supplementary Table 1 — Univariate survival analysis using a non-parametric test on each covariate in PSC cohort. PSC, pulmonary sarcomatoid carcinoma; A p-value of less than 0.05 represents a significant statistical difference. [file Table_1.docx]

**Table S1 Univariate survival analysis using a non-parametric test on each covariate in PSC cohort**

| Covariate | χ^2^ | P |
| --- | --- | --- |
| **Race** | 1.186 | 0.553 |
| White |  |  |
| Black |  |  |
| Others |  |  |
| **Sex** | 0.734 | 0.392 |
| Male |  |  |
| Female |  |  |
| **Region** | 1.015 | 0.798 |
| East |  |  |
| North |  |  |
| Southwest |  |  |
| Northwest |  |  |
| **Primary Site** | 14.648 | **0.023** |
| Upper lobe |  |  |
| Middle lobe |  |  |
| Lower lobe |  |  |
| NOS |  |  |
| Overlapping lesion |  |  |
| Main bronchus |  |  |
| Trachea |  |  |
| **Grade** | 8.444 | 0.077 |
| Grade I |  |  |
| Grade II |  |  |
| Grade III |  |  |
| Grade IV |  |  |
| Unknow |  |  |
| **Laterality** | 4.025 | 0.259 |
| Right |  |  |
| Left |  |  |
| Bilateral |  |  |
| Others |  |  |
| **Histological type** | 5.141 | 0.526 |
| NOS |  |  |
| Spindle |  |  |
| Giant |  |  |
| Small |  |  |
| Epithelioid |  |  |
| Undifferentiated |  |  |
| Desmoplastic |  |  |
| **Lymph nodes removed** | 55.734 | **<0.001** |
| 0-3 lymph nodes removed |  |  |
| ≥4 lymph nodes removed |  |  |
| Regional biopsy or aspiration |  |  |
| Sentinel |  |  |
| Others |  |  |
| **Radiation** | 11.366 | **0.01** |
| Beam radiation |  |  |
| Not specified |  |  |
| Unknown |  |  |
| Refused |  |  |
| **Chemotherapy** | 24.171 | **<0.001** |
| Yes |  |  |
| No |  |  |
| **Bone metastasis** | 6.202 | **0.045** |
| Yes |  |  |
| No |  |  |
| Other |  |  |
| **Brain metastasis** | 4.256 | 0.119 |
| Yes |  |  |
| No |  |  |
| Others |  |  |
| **Liver metastasis** | 10.038 | **0.007** |
| Yes |  |  |
| No |  |  |
| Others |  |  |
| **Lung metastasis** | 9.314 | **0.009** |
| Yes |  |  |
| No |  |  |
| Others |  |  |
| **First malignant indicator** | 8.504 | **0.004** |
| Yes |  |  |
| No |  |  |
| **Age at diagnosis (year)** | 28.230 | **<0.001** |
| <45 |  |  |
| ≥45, <55 |  |  |
| ≥55, <65 |  |  |
| ≥65, <75 |  |  |
| ≥75 |  |  |
| **Insurance status** | 5.193 | 0.158 |
| Medicaid |  |  |
| Insured or no specifics |  |  |
| Uninsured |  |  |
| Blanks or unknown |  |  |
| **Marital status** | 10.773 | **0.005** |
| Married or domestic partner |  |  |
| Divorced or separated or single or windowed |  |  |
| Unknown |  |  |
| **High school education (Score)** | 6.778 | 0.079 |
| ≤1000=1 |  |  |
| 1000-2000 |  |  |
| 2000-3000 |  |  |
| ≥3000 |  |  |
| **Median family income**  **($/month)** | 4.319 | 0.229 |
| ≤5000 |  |  |
| 5000-7000 |  |  |
| 7000-9000 |  |  |
| >9000 |  |  |

PSC, pulmonary sarcomatoid carcinoma;

A p-value of less than 0.05 represents a significant statistical difference.
